# Risk factors for clinical stages of COVID-19 amongst employees of the International Committee of the Red Cross (ICRC) worldwide over a period of 12 months

**DOI:** 10.1186/s12879-023-08674-2

**Published:** 2023-10-10

**Authors:** Maria Carla Tinti, Stéphanie Cécilia Guisolan, Fabrice Althaus, Rodolfo Rossi

**Affiliations:** International Committee of the Red Cross, 19, Avenue de la Paix, Geneva, 1202 Switzerland

**Keywords:** Circulating VoC, Humanitarian aid worker, Risk factor, SARS-CoV-2, Staff health, Workforce

## Abstract

**Background:**

Essential workers carry a higher risk of SARS-CoV-2 infection and COVID-19 mortality than individuals working in non-essential activities. Scientific studies on COVID-19 risk factors and clinical courses for humanitarian aid workers (HAW) specifically are lacking. The nature of their work brings HAW in proximity to various populations, therefore potentially exposing them to the virus. The objective of this study is to assess severity degrees of COVID-19 in relation to multiple risk factors in a cohort of HAW.

**Methods:**

Retrospective cohort study of data collected by the Staff Health Unit of the International Committee of the Red Cross, over 12 months (February 2021 – January 2022). Prevalence of demographic and health risk factors and outcome events were calculated. Factors associated with disease severity were explored in univariable and multivariable logistic regression models. Resulting OR were reported with 95%CI and p-values from Wald Test. P-values < 0.05 were considered significant.

**Results:**

We included 2377 patients. The mean age was 39.5y.o. Two thirds of the patients were males, and 3/4 were national staff. Most cases (3/4) were reported by three regions (Africa, Asia and Middle East). Over 95% of patients were either asymptomatic or presented mild symptoms, 9 died (CFR 0.38%). Fifty-two patients were hospitalised and 7 needed a medical evacuation outside the country of assignment. A minority (14.76%) of patients had at least one risk factor for severe disease; the most recorded one was high blood pressure (4.6%). Over 55% of cases occurred during the predominance of Delta Variant of Concern. All pre-existing risk factors were significantly associated with a moderate or higher severity of the disease (except pregnancy and immunosuppression).

**Conclusions:**

We found strong epidemiological evidence of associations between comorbidities, old age, and the severity of COVID-19. Increased occupational risks of moderate to severe forms of COVID-19 do not only depend on workplace safety but also on social contacts and context.

## Background

Essential workers - including those in healthcare, social and medical care, and transportation - carry a higher risk of SARS-CoV-2 infection and COVID-19 mortality than individuals working in non-essential activities [1–5]. However scientific studies on COVID-19 risk factors and clinical courses for humanitarian aid workers (HAW) specifically are lacking. The nature of their work brings HAW in proximity to various populations, therefore potentially exposing them to the virus. The risk is also increased by specific activities - as the ones in health settings - with a high percentage of potentially affected individuals.

COVID-19 has largely affected the International Committee of the Red Cross (ICRC) workforce. Data on COVID-19 cases within the ICRC workforce were collected from the onset of the pandemic to track cases and outcomes, to monitor the evolution in this specific population, and to inform the actions of the organisation to protect staff while maintaining operational continuity.

In March 2020, a crisis mechanism was activated within the ICRC, with the implementation of multiple measures: PPEs were distributed within the shortest possible delay, ECDC indicators of risk (incidence, mortality, basic reproductive number, etc.) [6] were adapted to ICRC context to advise on public health and social measures for the workforce, and once the WHO EUL vaccines [7] became available strategies to provide access to vaccinations were adopted.

The review of the factors associated with the development of different clinical stages of COVID-19 could provide a basis to improve preparedness in future similar scenarios.

The studied population also represents a specific group, working in various regions of the world with different levels of implementation of non-pharmacological infection control methods by the local health authorities, and with diverse COVID-19 epidemiological trends. We therefore aimed to assess severity degrees of COVID-19 in relation to various risk factors. While multiple studies have been published on the topic since the onset of the pandemic [8–12] none of them focused on the workforce of a humanitarian organisation. Moreover, most studies identified older age as one of the primary risk factors for severe disease, and young age as a protective factor [13] both of which are of less relevance to a working-age cohort. By excluding individuals over 65 and less than 20 we can potentially identify additional risk factors and/or confirm already known ones.

## Methods

A preliminary literature search was carried out via web-based search engines (Google Scholar and PubMed), using the following keywords (“COVID-19” OR “SARS-CoV-2”) AND (“humanitarian aid workers”) AND (“comorbidities” OR “hospitalisations”). To have a better understanding of the possible risk factors predicting disease severity, only meta-analysis and systematic reviews were selected given the vast amount of literature published since the onset of the pandemic. Snowball technique was used for reference chasing and tracking citations. Only resources published in English were considered. Publications not using original data (like editorials, opinion letters and grey literature materials) were excluded. We also excluded studies on children because they are not relevant to our research question [14–20].

The primary outcome of interest being the severity of the disease, the following items were considered : gender (male, female), age group (< 35, > 36 < 45, > 46 – categorized by using quartiles), type of contract (Geneva based for headquarters (HQ) staff, mobile for international recruited staff deployed in the field, resident for locally recruited staff deployed in their country of origin, “other” for cases for which the contract type did not fall in one of the other categories), region of work (ICRC internal classification identifies six different regions: HQ for Geneva, Africa, Asia-Pacific, Eurasia, Americas, Near and Middle East), work profile (i.e. health workers), place of treatment (inpatient/outpatient), health risk factors (high blood pressure HBP, obesity, chronic obstructive pulmonary disease COPD, history of ischemic heart disease IHD, asthma, diabetes, immunodepression, pregnancy, age).

We adapted the disease severity classification defined by the World Health Organization (WHO). COVID-19 infection was categorised into asymptomatic, mild, moderate, severe infection or death. Mild COVID-19 was defined as respiratory symptoms without evidence of pneumonia or hypoxia, while moderate or severe infection was defined by the presence of clinical and radiological evidence of pneumonia. Cases with SpO2 < 90% on room air or respiratory rate > 30 breaths/min were classified as severe [21].

The periods of diagnosis were used as proxy for the period of prevalence of the different COVID-19 variants of concern (VoC): from February 2021 to March 2021 for the Pre-Delta variants, from April 2021 to November 2021 for the VoC Delta, from December 2021 to January 2022 for the VoC Omicron [22, 23].

We conducted a retrospective observational cohort study using data collected by the Staff Health Unit of the ICRC in Geneva, Switzerland, over a period of 12 months (February 2021 – January 2022). The study population was represented by the entire ICRC workforce, affected by COVID-19, regardless of the country of assignment. Staff health personnel based in the countries were tasked with the collection of information that was communicated to a medical doctor in charge of data collection and patient monitoring in Geneva. The data were stored in a confidential database, accessible only to medical Staff Health personnel in Geneva.

The dataset was anonymised of all possible identifiers (e.g., date of birth was replaced by age in years) before being transferred to STATA 16 for statistical analysis. Categorical variables were numerically encoded, and continuous variables categorised. The prevalence of each risk factor and outcome events was calculated. The outcome of interest was dichotomised as mild (or less severe) and moderate (or more severe, including death). Factors associated with disease severity were explored in univariable and multivariable logistic regression models, and resulting odds ratio (OR) were reported with 95%CI and p-values from Wald Test. Only relevant risk factors were included in the multivariable analysis. We considered p-values < 0.05 as statistically significant and p-values between 0.05 and 0.1 as marginally significant.

## Results

The initial dataset included 2555 patients. After removing observations for which the COVID-19 level of severity was missing, the total sample for analysis was reduced to 2377 patients. The mean age was 39.5 years (Standard Deviation, SD = 9.9 years); the median age was 39 years (first and third interquartile 33 years and 46 years, respectively). Fifty-seven patients (2.4%) were aged 60 years old or above. Almost two thirds of patients were male and almost three quarters were national staff. About three quarters of cases were reported by three Regions (Africa, Asia & Western Pacific and Near & Middle East). Table 1 summarises the demographic characteristics of the ICRC COVID-19 patients.

**Table 1:** Demographic characteristics of patients

Variable (N)	n	%
**Gender (N = 2376)**		
Male Female	1472 904	61.95 38.05
**Age group (N = 2374)**		
≤ 35 years 36–45 years 46–59 years 60 + years	861 890 566 57	36.27 37.49 23.84 2.40
**Type of contract (N = 2372)**		
Geneva/mobile/other Resident (national)	614 1758	25.89 74.11
**Region of work (N = 2377)**		
HQ* Africa Asia/Pacific Europe/Central Asia Americas Near/Middle East	66 602 548 329 179 653	2.78 25.33 23.05 13.84 7.53 27.47
**Work profile (N = 2377)**		
Health staff Other	56 2321	2.36 97.64

* includes ICRC staff working in support centres of Manila and Belgrade

Over 95% of patients were either asymptomatic or presented mild disease, while 9 died (CFR 0.38%). Fifty-two (2.25%) were treated in hospital and 7 needed medical evacuation outside the country of assignment. One in seven patients (256, 14.76%) had at least one risk factor for severe disease; the risk factor most recorded was high blood pressure in 4.6% of the whole cohort. Over 55% of cases occurred during the predominance of Delta as the circulating VoC. Table 2 summarises the clinical characteristics and risk factors of patients.

**Table 2:** COVID-19 clinical characteristics, risk factors and circulating VoC

Variable (N)	n	%
**Severity degree (N = 2377)**		
Asymptomatic Mild Moderate Severe Death	241 2037 73 17 9	10.14 85.70 3.07 0.72 0.38
**Place of treatment (N = 2297)**		
Not in hospital In hospital	2245 52	97.74 2.26
**Medical evacuation (N = 2377)**		
Yes No	7 2370	0.29 99.71
**Number of risk factors (N = 2377)**		
Zero One Two Three or more	2112 223 35 7	88.85 9.38 1.47 0.29
**Specific risk factors (N = 2377)**		
Asthma COPD High Blood Pressure History of IHD Diabetes Obesity	43 8 110 10 54 62	1.81 0.34 4.63 0.42 2.27 2.61
Immunodepression Pregnancy	10 18	0.42 0.76
**Estimated circulating VoC (N = 2387)**		
Pre-Delta Delta Omicron	318 1313 741	13.41 55.35 31.24

In the univariate analysis, various known factors were associated with having a moderate or higher severity disease. Age (cOR = 1.41, 95%CI 1.18–1.79), Delta and pre-Delta circulating VoC compared to Omicron (cOR = 0.32, 95%CI) and pre-existing risk factors (cOR = 2.43, 95%CI 1.78;3.30). ICRC staff on resident contract had 83% (cOR = 1.83, 95%CI 1.06–3.15) higher odds of developing moderate or higher severity of COVID-19 compared to all other types of ICRC contracts. Health staff had higher odds compared to other staff profiles of moderate or higher severity of disease, but this association was marginally significant (p = 0.079). Gender and region of work were not associated with moderate or higher severity of disease.

All pre-existing risk factors were significantly associated with moderate or higher severity of the disease (except pregnancy and immunosuppression). The odds of moderate or higher severity of the disease were linearly associated with the number of risk factors (aOR for linear trend = 2.14, 95%CI 1.56;2.96). Those with the strongest association were COPD (cOR = 7.81, 95%CI 1.56;39.18), history of ischemic heart disease (cOR = 5.85, 95%CI 1.23;27.92), diabetes (cOR = 4.27, 95%CI 1.96;9.30) and obesity (cOR = 4.20, 95%CI 2.00;8.78).

The multivariable analysis showed very similar results to those of the univariate analysis. Table 3 summarises the results of the univariable and multivariable logistic regression analysis.

**Table 3:** Factors associated with having moderate (or higher severity) against having asymptomatic infection or mild disease. Results for univariate and multivariate logistic regression

Variable	Crude OR (95%CI)	p-value*	aOR (95%CI)°	p-value*
**Age group**				
≤ 35 years	Ref	—	Ref	—
36–45 years	1.26 (0.76;2.11)	0.368	1.22 (0.72;2.05)	0.461
46–59 years	1.79 (1.06;3.03)	0.030	1.61 (0.93;2.80)	0.089
60 + years	3.63 (1.44;9.20)	0.006	2.82 (1.02;7.79)	0.046
Linear trend	1.41 (1.18;1.79)	0.004	1.32 (1.05;1.69)	0.029
**Gender**				
Male	0.90 (0.60;1.36)	0.622		
Female	Ref	—		
**Type of contract**				
Geneva/mobile/other	Ref	—	Ref	—
Resident	1.83 (1.06;3.15)	0.030	1.88 (1.06;3.31)	0.030
**Region of work**				
HQ#	Ref	—		
Africa	2.35 (0.31;17.75)	0.408		
Asia/Pacific	2.21 (0.29;16.81)	0.445		
Europe/Central Asia	1.83 (0.23;14.68)	0.570		
Americas	3.04 (0.37;24.79)	0.299		
Near/Middle East	4.47 (0.60;33.00)	0.142		
**Circulating VoC**				
Pre-Delta	Ref	—	Ref	—
Delta	0.98 (0.57;1.69)	0.948	1.04 (0.59;1.83)	0.894
Omicron	0.32 (0.15;0.66)	0.002	0.37 (0.17;0.78)	0.010
**Work profile**				
Health staff	2.32 (0.91;5.95)	0.079	2.43 (0.89;6.62)	0.082
Other	Ref	—	Ref	—
**Number of risk factors**				
None	Ref	—	Ref	—
One	1.99 (1.12;3.52)	0.019	1.96 (1.09;3.51)	0.024
Two	8.16 (3.59;18.57)	< 0.001	5.52 (2.29;13.32)	< 0.001
Three or more	11.02 (2.10;57.71)	0.005	7.83 (1.44;42.50)	0.017
Linear trend	2.43 (1.78;3.30)	< 0.001	2.14 (1.56;2.96)	< 0.001
**Presence of risk factors**				
Any	3.50 (2.28;5.35)	< 0.001		
HBP	2.45 (1.24;4.85)	0.010		
Obesity	4.20 (2.00;8.78)	< 0.001		
COPD	7.81 (1.56;39.18)	0.013		
History of IHD	5.85 (1.23;27.92)	0.027		
Asthma	2.42 (0.85;6.90)	0.099		
Diabetes	4.27 (1.96;9.30)	< 0.001		
Immunodepression	2.57 (0.32;20.51)	0.372		
Pregnancy	1.36 (0.18;10.30)	0.768		

* p-value from Wald Test

° the multivariable model included age, type of contract, circulating VoC, work profile, number of risk factors

# includes ICRC staff working in support centres of Manila and Belgrade

## Discussion

To the best of our knowledge, this has been one of the few studies exploring the risk factors of COVID-19 clinical stages amongst HAW. We evaluated the data collected over a period of 12 months and identified that gender was not associated with a higher risk of having a severe form of the disease with an unfavourable prognosis. This is in accord with the studies published so far on this topic [8, 9]. Older age (46–59 and 60 + group) was significatively associated with the disease severity: this confirms the outcomes and results found by multiple authors and the model-based analysis by Verity et al. [24].

In addition to epidemiological factors, comorbidities also affected the disease severity and prognosis. In our population, obesity and diabetes predominantly contributed to the disease severity and were associated with a worse prognosis, confirming previous literature findings [20, 25, 26]. We also found that hypertension was associated with a higher severity of the disease and all prognostic endpoints, as well as COPD. However, this should be interpreted with caution since, in general terms, ICRC staff might have a different profile from the general population – given the need to undergo a medical screening prior to employment. This screening evaluates the “fitness to work” based on specific criteria, selecting healthier workers (i.e., the concept of the “Health worker effect phenomenon”), which might explain the reduced sample size of staff affected by the above-mentioned risk factors [27].

Our analysis confirmed the association between the direct exposure linked to the profession of humanitarian aid workers in the health field compared to other staff profiles with a moderate or higher severity of disease. This association was only marginally significant in the population we studied.

Given the geographical distribution of our workforce, we used the duty station of staff as a criterion to search for any relationship between the severity of the disease and the location of the patient but failed to identify any: region of deployment was not statistically associated with severity of disease.

On the contrary a strong association was found between the type of contract and the clinical stage of the disease developed. ICRC workforce can be divided in three main categories: staff working at headquarters, international staff working in the field, and national staff hired and working in their country of residence. Our data shows that staff on resident contracts have been more affected by COVID-19 and have been more at risk of developing more severe forms of the disease. Given that the organisation put in place the same preventive and protective measures at the workplace for the global workforce, higher level of infection and severity amongst staff on a resident contract might not be dependent on this but on other factors, like social interactions outside the workplace or lower levels of implementation of preventive measures in the private environment. The degree of severity of the disease could be linked to pre-existing risk factors (single or multiple) in this specific population, factors that could not be assessed in this study. These findings might be interesting to evaluate possible improvements to health screenings or the development of awareness campaigns.

Our study provides a unique overview of the prognosis-related factors affecting HAW during the COVID-19 pandemic, and how they influenced the severity of the disease. Pre-existing risk factors and age groups remain the most relevant prognostic indicators of the evolution of the disease. Moreover, the large sample size conferred significance to most of the risk factors identified.

The findings are affected by some limitations: first, although we proceeded with a systematic collection of the data, not all cases were reported or adequately registered, thus causing some loss of data. Second, the parameters were set at an early stage of the COVID-19 pandemic and some indicators might not have been included; this is in part due to the dataset being created for individual medical follow-ups and not with an aim of research. Third, we could not analyse the impact of vaccines on the clinical stages of COVID-19 because disparity in vaccine availability across the world meant ICRC could not implement a vaccination policy (mandatory completion of primary cycle of COVID-19 vaccine with a WHO EUL product) before November 2021, with effects only observable as of January 2022. Likewise, calendar periods were used as proxy for circulating VoC; while this proxy may be reliable for some context with high genomic sequencing capacity, it may not be so for other contexts. Due to the reasons above, we could not ascertain whether the lower severity seen during the Omicron circulating VoC was due to this VoC or the beginning of access to vaccination for our staff. Thus, these results should be interpreted with caution. Given the available data on exposures, residual confounding cannot be ruled out.

## Conclusions

Our study highlighted that comorbidities and old age show strong epidemiological evidence of association with the severity and prognosis of COVID-19 in a HAW population. Furthermore, staff on a resident contract were more affected than other categories of staff despite identical preventive measures being implemented at the workplace. These differences suggest that increased occupational risk of moderate to severe forms of COVID-19 does not only depend on workplace safety but also on social contacts and context. Collaborative actions must remain in place to guarantee that all preventive measures are adequately implemented and followed to reduce the risks related to COVID-19 infection in a future where humanity is going back to a new normal.

We have identified three main pillars to better protect HAW and respond more efficiently in case a similar scenario should repeat itself in the future: the implementation of a surveillance system for early identification of potential infective threats, the necessity to establish a PPEs stock – preferably in regional hubs – based on the epidemiological risk profile of the area and the need to scale up the capacity to deliver them in places that are not easily accessible, and the availability of actionable policies (e.g. within our organisation a mandatory COVID-19 vaccination policy came into force in November 2021, this could have been anticipated based on the vaccine availability trends in 2021). The presence of a medical service within the organisation, and medical screening procedures for workforce should also be considered as elements in favour of a better handling of individual cases and possibly a good tool for limiting severe and fatal cases - ensuring a rapid reaction to medical situations and a population with better basic health.

## Data Availability

The data that support the findings of this study are available from the corresponding author, MCT, upon reasonable request.
